# Accidents and Emergencies

**Published:** 1893-03

**Authors:** 


ACCIDENTS AND EMERGENCIES. A Manual of the Treatment of Surgical and Medical Emergencies in the Absence of a Physician. By CHARLES W. DULLES, M. D., Fellow of the College of Physicians, Philadelphia, and of the Academy of Surgery; Physician to the Rush Hospital; formerly Surgeon to the Out-door Department of the Hospital of the University of Pennsylvania, and of the Presbyterian Hospital, in Philadelphia, and Assistant Surgeon, Second Regiment, N. G., Pa. Fourth edition, thoroughly revised and enlarged, with new illustrations. Philadelphia: P. Blakiston, Son & Co., No. 1012 Walnut street. 1892.

That this little book has reached a fourth edition speaks for itself. The author sounds the key-note when, in his preliminary remarks, he warns the laity not to assume too much medical knowledge, and thus be led into doing more than is judicious for the patient. He says: "The true principle is, when urgency is pressing, to do what is known to be helpful; and when one is not sure, to do nothing." In this particular the book throughout is quite consistent, for scarcely anywhere is advice given which may not be rightly carried out by the intelligent bystander. The author considers all the usual emergencies, such as obstructed respiration, foreign bodies, unconsciousness, fits, burns, frost-bites, sprains, luxations, fractures, wounds, poisons, and the common diseases with urgent symptoms. There are throughout the book little "kinks" which one does not usually meet with in brochures of this kind, such, for instance, as (page eighteen) blowing in the ear to dislodge foreign bodies in the throat; (page twenty-three) the use of olive or castor oil in retained foreign bodies in the eye; (page thirty) in hysteria the eye-balls are persistently rolled up, and so on. The author also has opinions of his own, as evidenced by his treatment of frost-bite (page forty-seven), and general freezing of the body. The immediate use of heat recommended corresponds exactly with our own experience, and gives one hope that the older method will be abandoned for the new.

The book is a first-class one to have in a home, and every intelligent reader must be benefited by the lessons taught. The publishers have made this little volume most attractive, the type and general arrangement being of superior order. J. P.

